# Large-Scale Simulation of Full Three-Dimensional Flow and Combustion of an Aero-Turbofan Engine on Sunway TaihuLight Supercomputer

**DOI:** 10.3390/e25030436

**Published:** 2023-03-01

**Authors:** Quanyong Xu, Hu Ren, Hanfeng Gu, Jie Wu, Jingyuan Wang, Zhifeng Xie, Guangwen Yang

**Affiliations:** 1 Institute for Aero Engine, Tsinghua University, Beijing 100084, China; 2 National Supercomputing Center in Wuxi, Wuxi 214072, China; 3 Department of Energy and Power Engineering, Tsinghua University, Beijing 100084, China; 4 School of Aerospace Engineering, Tsinghua University, Beijing 100084, China; 5 Department of Computer Science and Technology, Tsinghua University, Beijing 100084, China

**Keywords:** whole aeroengine simulation, three-dimensional flow, combustion, sprayDyMFoam, Sunway TaihuLight supercomputer

## Abstract

Computational fluid dynamics- (CFD-) based component-level numerical simulation technology has been widely used in the design of aeroengines. However, due to the strong coupling effects between components, the numerical simulation of the whole engine considering the full three-dimensional flow and multi-component chemical reaction is still very difficult at present. Aimed at this problem, an efficient implicit solver, ‘sprayDyMFoam’ for an unstructured mesh, is developed in this paper based on the Sunway TaihuLight supercomputer. This sprayDyMFoam solver improves the PIMPLE algorithm in the solution of aerodynamic force and adjusts the existing droplet atomization model in the solution of the combustion process so as to meet the matching situation between components and the combustion chamber in the solution process. Meanwhile, the parallel communication mechanism of AMI boundary processing is optimized based on the hardware environment of the Sunway supercomputer. The sprayDyMFoam solver is used to simulate a typical double-rotor turbofan engine: the calculation capacity and efficiency meet the use requirements, and the obtained compressor performance can form a good match with the test. The research proposed in this paper has strong application value in high-confidence computing, complex phenomenon capturing, and time and cost reduction for aeroengine development.

## 1. Introduction

With the development of computational fluid dynamics (CFD), numerical simulations have been broadly employed in aeroengine design and development. The application of numerical simulation technology has many advantages, including effective enhancement of the design precision, reduction of the experimental iterations and development costs, shortening the development cycle, and improvement of the efficiency and quality of research [1,2,3]. At present, component-level simulation technology has been extensively utilized in component performance evaluation and optimization design, which effectively promotes the improvement of component design levels. However, for the whole performance analysis of the aeroengine with multiple components and multiple physical and chemical processes, there exists a fairly strong coupling between components [4,5]. As a result, the performance simulation of the whole engine still confronts major challenges in terms of the parallel algorithm efficiency of numerical solvers and supercomputing resources.

The performance simulation of aeroengines based on three-dimensional CFD is receiving more and more attention. The Center for Turbulence Research (CTR) [6] at Stanford University is the first one to carry out a full three-dimensional unsteady CFD simulation. Based on interface technology, the switch to Reynolds-averaged Navier-Stokes (RANS) simulations and large eddy simulations (LES) among three main components has been comprehended, and the complete simulation of the full engine has been realized, which verifies the feasibility of the numerical testbed based on the three-dimensional CFD. Based on the circumferential mean volumetric force model and the steady-state combustion simulated by RANS, and through the unidirectional coupling of component boundary conditions, Turner (2010) from the University of Cincinnati has also achieved a whole engine simulation with a low computational cost and reported that the error of the simulation results would be lower than 4% [7]. Wang (2013) from Imperial College London cooperates with Rolls-Royce to solve RANS equations of the S2 plane of the engine based on AU3X code, in which the combustor and air system utilize an unstructured grid, and the errors of the total temperature and pressure fields on the key section were less than 5% [8]. Krivcov et al. (2014) from the State University of Aeronautics and Astronautics, Samara, Russia, implemented ANSYS CFX to simulate the performance of a small gas turbine engine and compared the results with Astra one-dimensional simulation software to check the consistency of the two models for the engine performance [9]. Teixeira et al. (2018) from Numeca International in Brussels have performed a single-channel RANS machine simulation for micro-engines by utilizing two rotostatic interface processing methods of mixing surface and a nonlinear harmonic method [10]. One of the main goals was to lessen the calculation cost; however, part of the calculation precision was sacrificed. The simulation results of the machine according to the aforementioned approaches were then compared. Based on the AVBP unstructured grid solver, the European Center for Scientific Computing collaboration with Safran [11], completed the first full-cycle LES coupling calculation of the engine high-pressure compressor and combustor. For this purpose, 1500 million cells were used to realize the high-precision three-dimensional simulation of the whole aero-engine on a very large scale. However, three-dimensional full-cycle aerodynamic and combustion calculations of the whole engine, including fans, low-and high-pressure compressors, combustors, and low- and high-pressure turbines, have not been performed yet. Zhang et al. (2020) of the Gas Turbine Research Institute in China carried out a three-dimensional simulation of the core engine of the aeroengine in consideration of complex boundary conditions such as turbine blade cooling and verified the accuracy of a three-dimensional simulation of a whole aeroengine by comparing the performance parameters of the components with the experimental results, but the axial work of the turbine and the compressor deviated from the design balance point by 5.5% and 1.8% respectively [12].

This paper presents a new solver, the sprayDyMFoam solver, which is modified by our team based on the OpenFOAM code [13]. With the arbitrary mesh interface (AMI) boundary processing mechanism, programming wall barriers were broken, and the parallel ability was improved effectively; meanwhile, supercomputer resources were optimized reasonably. These technological innovations have made three-dimensional simulations of the whole aeroengine a reality [14].

From the high-performance computing (HPC) perspective, this work is the first effort to efficiently scale an implicit solver for an unstructured mesh to over four million cores on a heterogeneous supercomputer. While we already see highly efficient implicit solvers on the Sunway TaihuLight for structured mesh, the unstructured patterns in both memory and computation have apparently brought the challenge to the next level.

From the CFD standpoint, this is also a milestone on the Sunway TaihuLight, to support real-scenario three-dimensional aeroengine simulations. The largest scale simulation describes the whole engine with up to 5.1 billion unstructured mesh grids, with a fine capability to capture highly turbulent mix and reaction phenomena.

## 2. Current State of the Art

### 2.1. Simulation of the Aeroengine

As one of the most important components of the aeroengine, the aeroengine simulation has been a key research topic for both industrial and academic researchers.

Table 1 presents some of the significant existing efforts on this front. Concerning the scientific efforts that try to capture the mechanical behavior of jet flames, we see efforts that employ a Cartesian grid with around 500 million points and around 65,536 MPI (message-passing interface) processes. For industrial efforts to simulate combustors, we see that the problem mesh size has gradually increased from around 20 million points to around a few billion points, all utilizing an unstructured mesh to appropriately cover the complex geometries.

The reasons behind the limited growth of both parallel scale and problem mesh size are manifold and hard to summarize. One leading factor may come from the solver. The industrial aeroengine/gas turbine performance simulations may require more sophisticated and well-validated fluid and combustion models and, more importantly, dedicated optimizations on ultra-scale parallel computing to take advantage of the HPC resources available. Another non-negligible factor is the lack of feasible industrial pre- and post-processing tools for simulations in which mesh size and parallel scale are extremely large. As computer capabilities still remain promising to RANS, particularly in the industrial context, works done to bridge the powerful modern supercomputing resources to industrial engine performance simulation are far from sufficient. This issue also represents the main motivation that we try to make exemplary progress with present works.

In the full-circle three-dimensional calculation, in addition to combustion, it is also required to simulate the flow in the compressor and turbine by considering the matching situation between these components and the combustor. In order to accurately simulate flow and combustion and avoid interpolating between interfaces for various solvers, we employ the same unique solver. Therefore, while the solver has high-precision calculation capabilities, it also needs to have the functions of processing rotating parts, dynamic and static boundaries, and combustion. In addition, given a large amount of computer simulation calculations, the solver needs to have high-efficiency and large-scale parallelism. The sprayDyMFoam is an unsteady compressible solver that supports laminar and turbulent flows, moving mesh, and atomized combustion and can generally meet the requirements of full-cycle aeroengine simulations.

The fuel atomization that occurs in the combustor is a very vital process of combustion. The simulation accuracy of the atomization process directly affects the engine simulation accuracy. In the simulation of the atomization process, sprayDyMFoam can utilize the primary atomization model, such as hollow cone and solid cone, the secondary droplet breaking model, such as ReitzDiwakar, PlichErdman, ReitzKHRT, and the liquid phase heat exchange and evaporative phase change models. After the fuel is atomized, the eddy dissipation concept (EDC), partially stirred reactor model (PaSR), turbulent combustion model, or fast single-step reaction model can be employed to simulate the combustion process.

The flow field in the turbofan engine is tremendously complicated. It is a typical turbulent flow, so the solver must appropriately function based on a reasonable turbulence model to achieve more precise results. The sprayDyMFoam solver can utilize RANS turbulence models such as Spalart-Allmaras (S-A), k-epsilon (KE), and shear stress transport (SST). To handle steady-state flows and for the presence of separated flows, Smagorinsky, wall-adapting local eddy viscosity (WALE), and other LES-based models can be employed to enhance the simulation accuracy to ensure that the flow details are not overcome by numerical dissipation.

The governing equations associated with sprayDyMFoam can be written as:

(1) $$\begin{array}{l} \frac{\partial \rho }{\partial t}+\nabla \cdot \left(\rho U\right)=S_{parcels}^{\rho }\\ \frac{\partial \rho U}{\partial t}+\nabla \cdot \left(\rho UU\right)-\nabla \cdot \tau =-\nabla p+\rho g+S_{parcels}^{U}\\ \frac{\partial \rho h}{\partial t}+\nabla \cdot \left(\rho Uh\right)+\frac{\partial \rho K}{\partial t}+\nabla \cdot \left(\rho UK\right)-\nabla \cdot \left(\alpha_{eff}\nabla h\right)\\ =-\frac{\partial p}{\partial t}+\rho U\cdot g+S_{parcels}^{h}+S_{combustion}^{h}\\ \frac{\partial \rho Y_{i}}{\partial t}+\nabla \cdot \left(\rho UY_{i}\right)-\nabla \cdot \left(\mu_{eff}\nabla Y_{i}\right)=S_{parcels}^{Y_{i}}+S_{combustion}^{Y_{i}} \end{array}$$

where *U* is the velocity vector (m/s), *g* is the gravity vector (m/s^2^), *p* is the pressure (Pa), *ρ* is the density (kg/m^3^), *τ* is the shear stress (Pa), *h* is the enthalpy (J), *K* = 0.5|*U*|^2^, *α_eff_* is the effective thermal conductivity W/(m·K), *μ_eff_* is the effective viscosity coefficient kg/(m·s), *Y_i_* is the component mass fraction, and *t* is time (s). *S_parcels_* and *S_combustion_* are the atomized droplet particle phase source term [19] and combustion source term [20], respectively, and the superscripts *ρ*, *U*, *h*, *Y_i_* represent the source term of density, velocity, enthalpy, and component. In order to close the equation, we introduce the assumption that each component gas is an ideal gas, and the state equation of multi-component ideal gas is as:

(2) $$p=\sum p_{i},\rho =\sum \rho_{i},p_{i}=\rho_{i}\frac{R}{M_{i}}T$$

where *R* denotes the universal gas constant (J/(mol·K)), *M_i_* being the molecular weight (g/mol). 

The sprayDyMFoam solver adopts a pressure-based algorithm to solve the above control equations and employs PIMPLE iteration to deal with unsteady calculations. Among them, PIMPLE outer iteration is utilized to eliminate errors introduced by the linearization of the control equations; through momentum estimation and multi-step pressure correction methods, internal iteration can synchronize momentum and continuous equations. 

In the case of transonic flow, the momentum prediction equation and pressure diffusion equation can be utilized to enhance the calculation accuracy and stability. For the case of low-speed flow, turning off momentum prediction and exploitation of the pressure, Poisson’s equation can improve the calculation speed while ensuring the correct calculation of the flow field. Therefore, according to the actual flow situation, the sprayDyMFoam solver automatically sets the momentum estimation and control parameters of the transonic flow; thereby, it has better working condition adaptability.

Compared with the existing efforts, our work does manage to enhance both the parallel scale and problem mesh size to the next level. Our solver can function with an unstructured mesh with over 5.1 billion grids and can efficiently exploit more than 4.25 million cores of Sunway TaihuLight.

### 2.2. PDE Solvers

Problems based on partial differential equations (PDEs) are generally among the top popular issues for HPC systems to resolve for both scientific and engineering purposes. Table 2 highlights some of the most successful efforts selected as Gordon Bell Prize winners or finalists over the past decades in designing different PDEs solvers.

In the early decades, when multi-core CPUs were still the fastest supercomputers in the world, many successful works efficiently utilized implicit solvers with tens of thousands of cores [21,22,23,26].

Around the year 2010, we started to observe a clear diversion from homogeneous systems to heterogeneous ones with many-core accelerators, such as GPU or Intel MIC. Sunway TaihuLight is another type of the latter system that fuses both management and computing cores into a single chip.

As these many-core accelerators demonstrate a natural preference for compute-intensive patterns, in the early stage, most extreme-scale simulations would adopt explicit approaches to better utilize the cores [24,25]. On the other hand, implicit solvers, with more challenging patterns in both computation and memory access, involve more difficulties in achieving a balanced design that can efficiently exploit the many-core architectures. Only until the year 2016, we commenced seeing efficient implicit solvers that can employ various kinds of cores on a large scale. Yang et al. (2019) managed to utilize the 10 million cores of Sunway TaihuLight for one fully-implicit solver that tries to analyze the atmospheric dynamic equations on a global scale with a structured mesh [27].

Compared with the efforts mentioned above, this paper takes an even more tough challenge, with the goal of designing an efficient implicit solver for an unstructured mesh on heterogeneous supercomputers, such as Sunway TaihuLight. To the best knowledge of the authors, this is possibly one of the first attempts to exercise such an implicit approach for industrial design problems (aeroengine), which generally necessitate an unstructured mesh to describe the complex geometries of the target object.

## 3. Sunway TaihuLight and the Innovative Methods

### 3.1. System Overview of Sunway TaihuLight

The Sunway TaihuLight is a 40-rack Chinese homegrown supercomputing system with 40,960 compute nodes, providing a peak performance of 125 Pflops and a sustained Linpack performance of 93 PFlops. There is a unique design for the network topology of Sunway TaihuLight: supernode. One supernode is composed of 256 processors, and all processors within a supernode are fully connected by a customized network switchboard, enabling highly efficient all-to-all communications. The network topology for connecting all the supernodes is a two-level fat-tree using a customized high-speed interconnect network. The network link bandwidth is 16 GB/s, and the bisection bandwidth reaches 70 TB/s.

The software system of Sunway TaihuLight is developed to adapt the SW26010 processor, including customized 64-bit Linux OS kernel, global file system, compliers, job management system, etc. The Sunway compiling systems support C/C++ and Fortran, as well as mainstream parallel programming standards such as MPI and OpenACC. A lightweight thread library Athread is also provided to allow programmers to perform finer-grained optimization.

### 3.2. The SW26010 Processor

**Figure 1:**
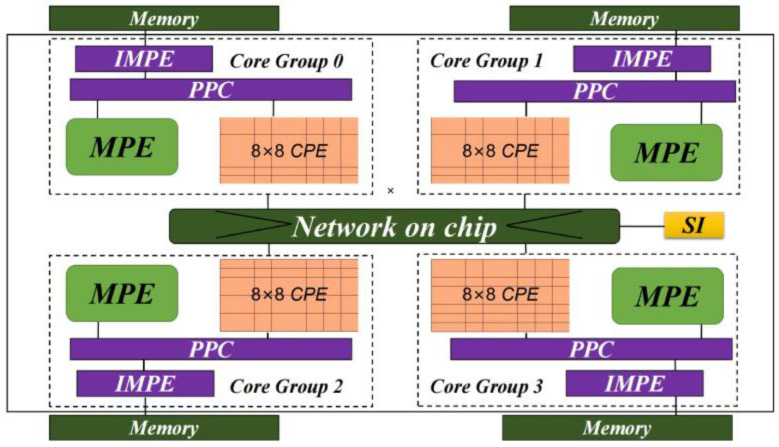
The Architecture of SW26010.

The SW26010 processor is designed by the Shanghai High-Performance IC Design Center. It is a many-core processor with 260 heterogeneous cores providing a peak performance of 3.06 TFlops and a performance-to-power ratio of 10 GFlops/Watt. As Figure 1 shows, the 260 cores are divided into four core groups (CGs). Each CG is composed of one management processing element (MPE), one intelligent memory processing element (IMPE), and 64 computing processing elements (CPEs) organized as 8 by 8 mesh. MPEs, IMPEs, and CPEs are designed for different goals. The IMPE has a single instruction fetch unit and multiple instruction buffers, mainly targeting memory access operations. The MPE is a complete 64-bit RISC core with a frequency of 1.45 GHz, mainly targeting handling the flow control of a program, I/O, and communication functions. While CPEs adopt a more simplified microarchitecture to maximize the aggregated computing power. Each CPE has a 16 KB L1 instruction cache and 64 KB local data memory (LDM), which can be configured as a user-controlled fast buffer. A performance model based on the three-level (REG-LDM-MEM) memory hierarchy was proposed by Fang et al. (2017) [28]. The CPE can either directly access the global memory with a limited bandwidth of 8 GB/s or through a REG-LDM-MEM memory hierarchy to obtain a much higher bandwidth. Each CPE has two pipelines to process instruction decoding and execution. A carefully orchestrated instruction reordering scheme can alleviate the dependencies of the instruction sequence, thus potentially improving the instruction execution efficiency. Inside each CPE cluster, a mesh network with 8 row communication buses and 8 column communication buses enables fast data communications and sharing at the level of registers, which allows efficient data reuse and communication among CPEs.

### 3.3. AMI Parallelization

Commonly, there exists a large number of grids for three-dimensional simulations. Additionally, there are many dynamic and static boundaries between multi-stage compressors and multi-stage turbines, and the usual AMI boundary parallel method has poor efficiency on supercomputers, which seriously affects the efficiency of large-scale parallel computing. Combined with the hardware environment of the Sunway supercomputer, the parallel communication mechanism of the AMI boundary processing is optimized, and the dynamic and static surfaces of multiple AMIs are combined to effectively solve the parallel bottleneck of the AMI. 

Taking the test of 40 million grid units as an example, with 1000 cores in parallel, AMI boundary interpolation coefficient calculation in a single time step takes 1300 s prior to the optimization procedure; nevertheless, it takes less than only 20 s after optimization. As a result, the AMI parallel optimization considerably improves efficiency.

Three-dimensional simulations are usually based on massively parallel computing; however, the usual fuel atomization Lagrangian module does not support the AMI boundary subdivision, influencing the implementation of massively parallel. Therefore, it is necessary to optimize the Lagrangian module, which mainly optimizes the processing rules for the movement of the fuel droplet to the AMI boundary. It implies that when the droplet moves to the AMI boundary, it is considered to have escaped the computational domain. Considering that the AMI boundary is all outside the combustor and the combustion reaction is only carried out in the combustor, nevertheless, this assumption will not affect the simulation results. After optimizing the droplet motion rules, the conflict between the large-scale parallelization and the Lagrangian module can be appropriately resolved. 

### 3.4. Performance Challenges

The data structure of the unstructured finite volume method (FVM) discretization can be regarded as an adjacency graph, with control volume cells as the vertices and faces between the edges of the cells. In the present solver, the topology of the FVM graph was stored with an index-saving format lower diagonal upper (LDU) to store the symmetry matrix. In industrial codes, numerical schemes with orders of no more than two are most frequently exploited, indicating low arithmetic density. According to the computation nature of unstructured FVM discretization, the data access from memory has to be irregular. In a parallel implicit solver, convergence, and parallelism must be carefully balanced to achieve the best performance. While combined with the ecosystem immaturity of the industrial aeroengine simulation on a supercomputer, the main performance challenge can be stated as:

memory bandwidth limited
irregular memory access
the tradeoff between parallelism and converging speed
large-scale aeroengine simulation toolchain shortage

### 3.5. A Customized Parallelization Design [29]

**Figure 2:**
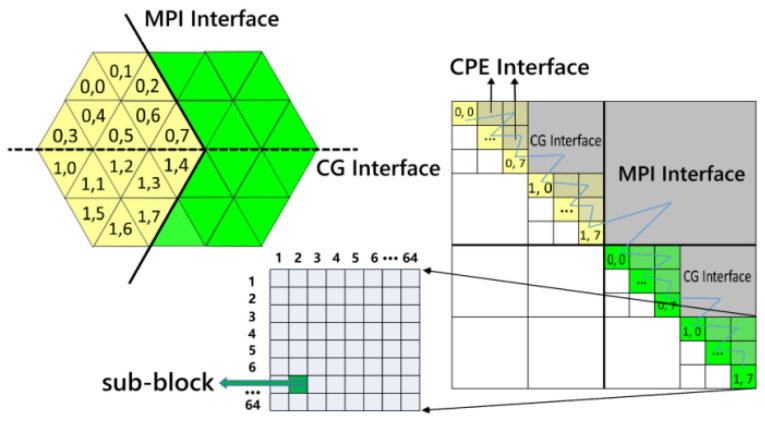
Multilevel Block reordering of unstructured mesh.

As mentioned above, the computational operation capability in the finite volume is extremely restricted by indirect and irregular addressing. Therefore, we propose a multi-level block (MLB) data structure and design a master-slave asynchronous parallel algorithm with the MLB, as demonstrated in Figure 2. The MLB format has a non-uniform multiblock structure based on an LDU matrix. The coarsest blocks represent the MPI level mesh decomposing. The coefficients on the coarse diagonal block indicate the internal faces of the mesh cells assigned to a process running on a single CG, and the off-diagonal coarse block denotes the MPI interface of cells that are connected but distributed on various processes. The medium blocks represent the segmentation of mesh corresponding to the total cache size of a CG, which makes it possible that the relevant data in unstructured mesh calculation is lumped together. At this level, all row blocks are marked as diagonal blocks, colored cells, and off-diagonal blocks, gray cells marked as the CG Interface. They are assigned to the CPEs and MPE, respectively, depending on the data density. To address the read-write conflict problem in the MPE-CPEs cooperated parallelism, we add a bias to the index of row blocks in the MPE relative to the CPEs.

Finally, the finest level decomposing parts would be confined to 64, corresponding to the number of the CPEs. The mesh data on the diagonal block is basically “local” for the cells assigned to a single CPE and directly loaded into the LDM by the DMA, while the off-diagonal block contains mesh data to be transferred by the on-chip register level communication. The blue filling curve represents the sequence of faces in different blocks. Generally, the priority rule is that the faces in the finer level block are prior to coarser ones, and in the same level, the row block is prior to the column block. Inside the leaf block, the face sequence is arbitrary. With the MLB format, unstructured data is now “block-structured,” and irregular data can be accessed continuously in a leaf block. Taking advantage of the graph partition tool Metis, most faces or coefficients will be gathered in diagonal blocks at each level, and the interfaces with “communication” will be minimized to a certain extent with load balance into consideration.

**Figure 3:**
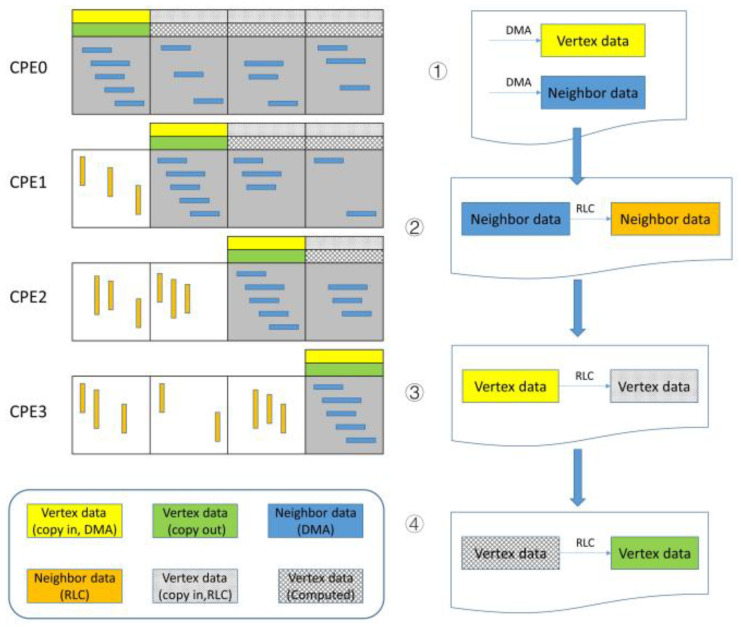
The computation process in CPEs.

As for the diagonal block in CPEs, one CPE is in charge of the computation of one sub-block row. To explain the computational pattern of slave cores, we take the Sparse Matrix-vector Multiply (SpMV) as an example. The coefficient matrix **A** is decomposed into three arrays, **lower**, **upper,** and **diag,** in LDU format, storing the lower triangle data, upper triangle data, and diagonal data, respectively. These data can be transferred into LDM completely through DMA because they are sparse. However, this pattern does not appropriate for **x** and **b** because they are dense. Figure 3 shows the computation process in CPEs, and the amount of CPEs is cut down to 4 for conciseness. In our implementation, the sub-block row is also divided into two parts: diagonal sub-block and off-diagonal sub-block. Firstly, we fetch **x** and **b**, namely VertexData in Figure 3, in the diagonal sub-block through DMA. The column index of all edges in the sub-row block is also fetched through DMA, namely NeighborData in Figure 3(①). Then we transfer the Neighbor Data from the upper triangle edges to the lower triangle edges through RLC, which is essential for the next step (②).

For the off-diagonal sub-block, though **x** cannot be loaded directly through DMA, they are stored in the LDM of other CPEs. Hence, **x** can be padded completely through RLC, while this part is stored sparsely, as well as edge data (③). The communication pattern is shown in Algorithm 1. Here, **SPE_NUMS** denotes the number of CPEs in one CG and is defined as a constant. The terms **total_send_pcg** and **total_recv_pcg** denote the count of send-packages and receive-packages in RLC for each CPE. The terms **sPacks** and **rPacks** belong to the **Packs** struct storing the package information in RLC, including **src_id**, **dst_id,** and **data**. Firstly, for every send-package, we can get the index of the edge through array **bias** and **edg_start**, storing the starting index of the edge of sub-blocks. Here the column index of edges, **Neighbor,** is transferred from other slave cores in the last RLC (②). Apparently, the **x** transferred through RLC is sparse and has the same length as edges in the off-diagonal sub-block. **reg_transfer_data()** performs the global on-chip register communication according to the struct **sPacks** and **rPacks**. In the last section, the existing data is padded with the **x** through RLC and then composes the completed **x**.
**Algorithm 1.** Register-Level Communication  1:  define SPE_NUMS 64  2:  **for** i = 1->SPE_NUMS **do**  3:    bias[i] = 0  4:  **end for**  5:  **for** ipcg = 1–>total_send_pcg **do**  6:    **if** (MYID> sPacks[ipcg].dst_id) **then**  7:        edge_id = edge_start[sPacks[ipcg].dst_id]  8:                      + bias[sPacks[ipcg].dst_id]  9:        sPacks[ipcg].data <– x[Neighbor[edge_id]] 10:      bias[sPacks[ipcg].dst_id]++ 11:    **end if** 12:  **end for** 13:  reg_transfer_data() 14:  **for** ipcg = 1–>total_recv_pcg **do** 15:    **if** (MYID > rPacks[ipcg].src_id) **then** 16:      x[length] <– rPacks[ipcg].data 17:      length++ 18:    **end if** 19:  **end for**

Now that the coefficient matrix **upper**, **lower**, **diag,** and vector **x** is loaded into LDM, we can perform the SpMV operation in the CPEs and get the output vector **b**. The storage pattern of **b** is identical to **x**. So as to avoid the write conflict between slave cores, **b** needs to be transferred to the corresponding CPEs through RLC (④). This RLC algorithm is similar to **x** but in the inverse direction. Finally, the output vector is transferred to MPE through DMA.

As mentioned above, the MLB decomposition level is estimated according to the LDM size by the next equation:

(3) $$levels\left[0\right]=\frac{\left(L_{e}C_{e}+L_{v}C_{v}\right)\;size\;of\;\left(scalar\right)+2\;size\;of\;\left(label\right)}{SPE_{NUM}size_{ldm}}$$

where *levels*[0] is the decomposition level, *L_e_* and *L_v_* are the lengths of edge and vertex respectively (m), *C_e_* and *C_v_* are the array number of edge and vertex, especially 3 and 2 for SpMV, and *scalar* and *label* are our alias of float and integer for the convenience of the type definition. *SPE_NUM_* and *size_ldm_* are the counts of slave cores in one CG and the size of LDM space, 64 and 65,536, respectively.

### 3.6. Efficient Linear Solving: Multilevel Parallelism

In our application, the systems of equations are analyzed by decoupling the variables: each equation is solved in a single matrix, and the inter-equation coupling terms are treated explicitly by exploiting existing values of unknowns. Various types of solution methods are chosen depending on the type of equations (i.e., hyperbolic, parabolic, or elliptic). The hyperbolic kind equations (velocity) are solved by employing Krylov methodologies like preconditioned bi-conjugate gradient stabilized method (PBiCGStab). The elliptic kind equations (pressure) are solved by utilizing the algebraic multigrid approach (AMG). The multilevel parallelism in both the MPIs and multicores is taken into account at the beginning of the linear solver design.

The AMG approach consists of describing the coarse grid, restriction operator, and coarse-grid operator and is smoother on each level. The coarsening algorithm exploits agglomeration to group multiple fine cells into a single coarse cell based on the face weights (face areas or matrix off-diagonal coefficients). The agglomeration AMG has been proven to be the most efficient methodology for solving the equation associated with the pressure field. The agglomeration results in a simple piecewise constant interpolation, and the coefficients on the coarse level matrix can be generated by directly summing coefficients on the fine level matrix. This kind of AMG scheme can be fully compatible with the MLB since it does not require an exchange of any information at the MPI level. Another crucial phase in solving is the smoother choice. The Gauss-Seidel approach is not the optimal choice for the SW26010 since it should be divided into 64 CPEs and is hard to parallelize due to its strong dependency. Hence, a polynomial smoother such as Chebyshev [30] is considered since it can be fully parallelized on the SW26010. The Chebyshev smoother needs to be aware of the largest of the matrix’s eigenvalues, which can be readily evaluated by implementing some simple diagonal preconditioned conjugate gradient (PCG) iterations [31]. Hence, the whole AMG scheme only consists of multiple SpMVs and vector operations (inner product, SAXPY), where these two phases have been able to be fully parallelized on the SW26010. The same principle is applied to the PBiCGStab method, where a diagonal (Jacobi) preconditioner is utilized.

From the MPI standpoint, the PCGs in the AMG and the PBiCGStab are both optimized by selecting algorithms that minimize the two global all-reduce operations [32].

### 3.7. Parallel Mesh Generation

**Figure 4:**
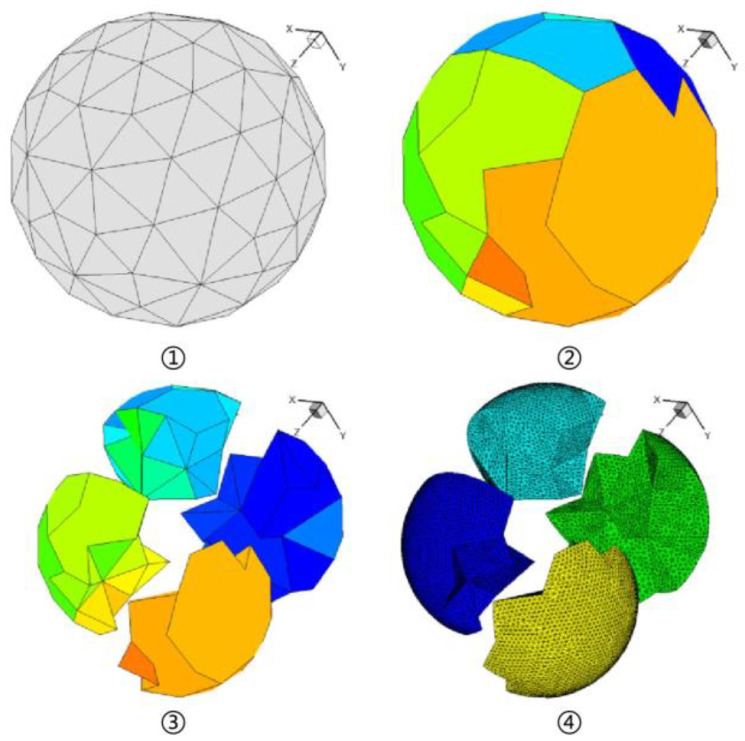
The procedure of shape-preserving parallel mesh generation.

In the present work, the 5.1 billion-size mesh generation is the first bottleneck encountered in the simulation procedure. Such a big mesh often takes terabytes of memory and storage, which is a painful task using a traditional sequentially running meshing tool. An in-house parallel unstructured meshing code is transplanted onto the Sunway TaihuLight and exploited to generate meshes of the aeroengine. Compared to mesh refining on coarse ones, details of the geometry are preserved as much as possible. The main procedure is demonstrated in Figure 4. The space is suitably meshed by a tree-like decomposing, and finally, the corresponding boundaries are re-meshed based on the input geometry to preserve the shape details [33]. The performed experiments and practices on the Sunway TaihuLight proved the capability of generating billions of mesh elements in dozens of minutes.

## 4. Performance Tests and the Results

### 4.1. Model and Settings

(a) Physics Runs

**Figure 5:**
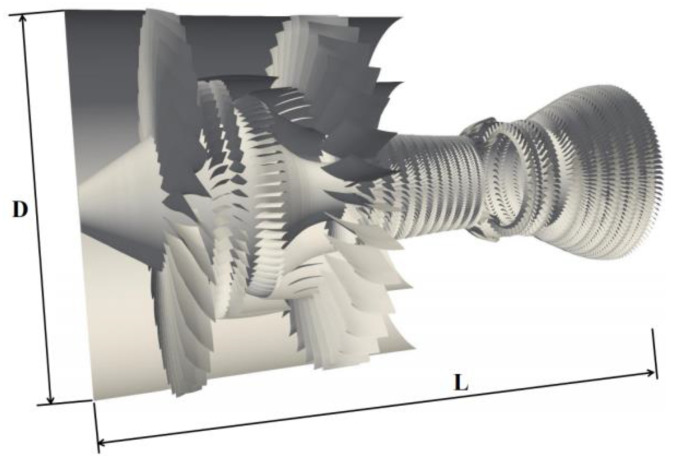
Schematic of the whole aeroengine used in this study. Fan tip diameter *D* = 2.11 m, axial length *L* = 3.42 m.

Figure 5 illustrates an aeroengine with two spools, two stages fan, a ten-stage compressor, a short annular combustor, and a seven-stage turbine. The performance is evaluated by simulating the three-dimensional unsteady flow and combustion of this whole aeroengine on the Sunway TaihuLight supercomputer based on the sprayDyMFoam solver. This physical run is designed to assess the accuracy of the solver by comparing it with the experimental data [34,35].

The boundary conditions are enforced as follows. The Mach number of the inlet flow is set equal to 0.5 (i.e., *Ma* = 0.5), and the Reynolds number is taken as *Re* = 2.12 × 10^7^, based on the diameter of the fan blade tip. The fan inlet conditions are defined as the total temperature, total pressure, and direction of the fluid flow. The outlet conditions of the fan and turbine are specified as static pressure. The no-slip boundary conditions are imposed on all solid walls. The fan and low-pressure turbine rotate at 3505 rpm, while the high-pressure compressor and turbine rotate at 12,290 rpm. The Euler implicit scheme with a fixed CFL number of 0.5 and an adjustable time step is exploited for the time advance solution, in which the Euler implicit scheme has the advantages of less computation and higher stability. The SST is chosen as the turbulence model, and the Abramzon-Sirignano model [36] is chosen as the evaporation model. The PaSR closure model [37] is adopted in the simulation.

**Figure 6:**
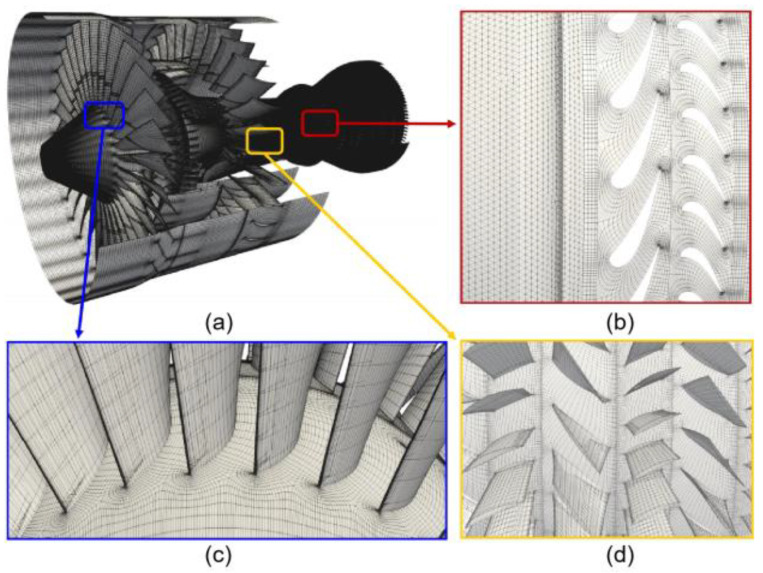
The coarse meshes of the whole aeroengine including fan, compressor, combustor, and turbine. The length of each element is about 1 × 10^−6^ *D*, in which *D* is the fan tip diameter. (**a**) Surface meshes of whole aeroengine, (**b**) Coarse meshes of combustor and turbine, (**c**) Coarse meshes of fan blades, (**d**) Coarse meshes of compressor blades.

Unstructured tetrahedral meshes for the combustor and hexahedral meshes for the remaining engine components are appropriately generated by utilizing the domain decomposition approach. All meshes have an identical isotropic resolution in their respective regions, and views of the coarse meshes of the whole aeroengine, including the fan, compressor, combustor, and turbine, are demonstrated in Figure 6.

In order to test the parallelism performance, various cases of the minimum mesh scale are designed, including CASE-1 to CASE-3. The mesh number of elements for each case is given in Table 3. The largest total element number appears in CASE-3, which is 5.1 billion. All runs are also performed under double-precision arithmetic.

(b) Peak Sustained Performance Runs

The peak sustained performance run was designed to evaluate the efficiency of this Large-scale simulation. The peak sustained performance run was performed using mesh CASE-3 on 65,336 SW26010 processors of Sunway TaihuLight.

(c) Scaling Runs

The scaling runs are designed to assess the scalability of this Large-scale simulation. Strong scaling concerns regarding the speedup for a fixed problem size with respect to the number of processors are commonly governed by Amdahl’s law [38]. Weak scaling concerns associated with the speedup for a scaled problem size with respect to the number of processors are governed by Gustafson’s law [39]. Both strong and weak scaling are tested on CASE-1, CASE-2, and CASE-3 separately with a series of incremental processes. The total number of time steps and current wall-clock time was then reported at each time step.

### 4.2. Performance Results

In this section, we illustrate the computational performance by simulating a full-size annular aeroengine as an example of a real-world application.

(a) Scaling

Scaling runs were undertaken to evaluate weak and strong scaling on the Sunway TaihuLight. Specifically, scaling runs on meshes CASE-1, CASE-2, and CASE-3 were employed to assess both weak and strong scaling.

The weak scaling results for the Sunway TaihuLight are provided in Table 4, where ncores represents the number of SW26010 cores, and *T_N_* denotes the runtime in seconds. Further, the strong scaling results for the Sunway TaihuLight are given in Table 5.

(b) Peak Sustained Performance

The peak sustained performance run is undertaken by implementing mesh CASE-3 on 4259840 SW26010 CPUs of the Sunway TaihuLight. The run had 35.7 billion DOFs and achieved 1384.92 DP-GFLOP/s.

(c) Physics

The physics run is undertaken with the mesh CASE-3 on 5000 SW26010 CPUs of the Sunway TaihuLight.

**Figure 7:**
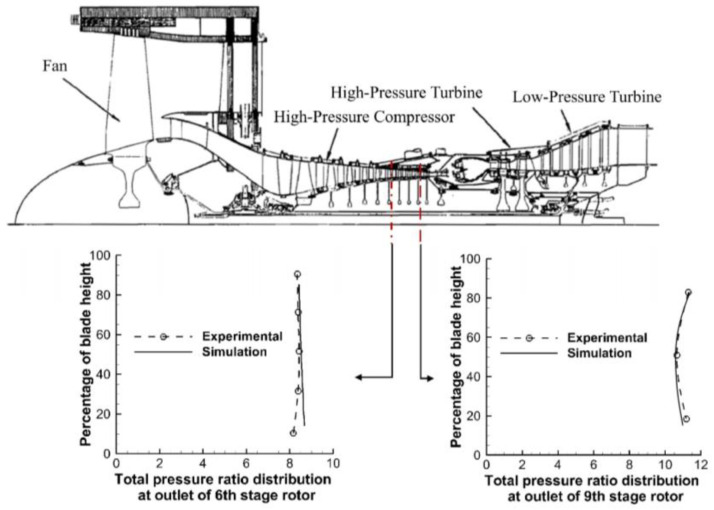
Simulation results and experimental data of total pressure distribution at the outlet of the 6th stage rotor of high-pressure compressor [34,35].

The simulated distribution of the total pressure ratio, along with the blade height from root to tip at the outlet of the 6th and 9th stage rotors of the high-pressure compressor, has been demonstrated in Figure 7. The predicted results are also compared with the experimentally measured values of the moving probe [34,35]. It is noticed that the results obtained by the sprayDyMFoam solver are in reasonably good agreement with the experimental data; thus, the accuracy of the calculation method and numerical model has also been verified.

**Figure 8:**
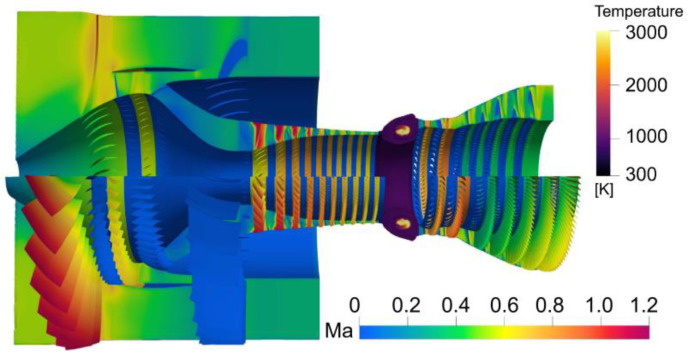
Simulation results of Mach number and temperature.

Figure 8 presents the simulation results of the Mach number and the temperature field in the whole aeroengine. The predicted results show the flow velocity distribution on the meridian section of the aeroengine, the speed of the rotor blades of the fan, compressor, and turbine, and the combustion phenomenon in the combustor.

**Figure 9:**
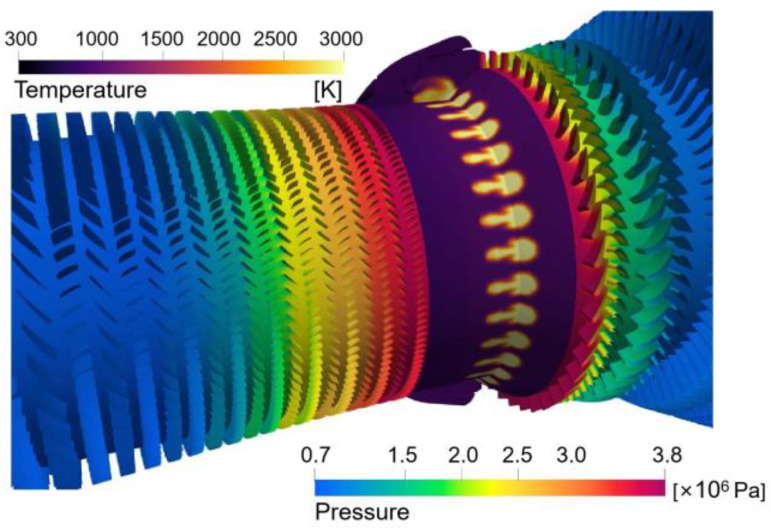
Simulation results of pressure and temperature.

Figure 9 demonstrates the simulation results of the pressure and temperature fields in the whole aeroengine. It can be seen from the plotted results that the pressure of the airflow is the highest at the outlet of the compressor through the work done by thousands of blades. The high-pressure gas is mixed with the fuel in the combustor, fully burns, and releases huge mechanical energy. The high-pressure, high-energy gas drives the turbine to do work. Subsequently, after passing the turbine, the pressure of the airflow decreases while the speed increases.

**Figure 10:**
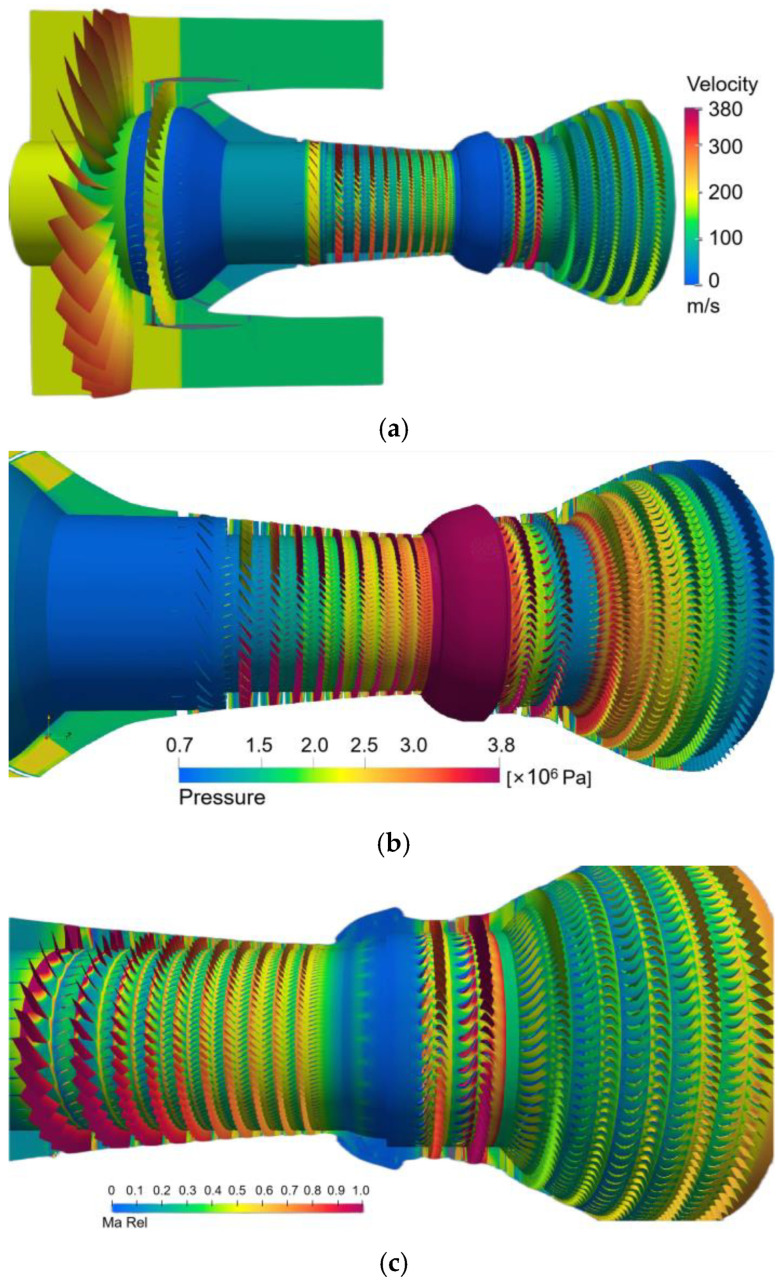

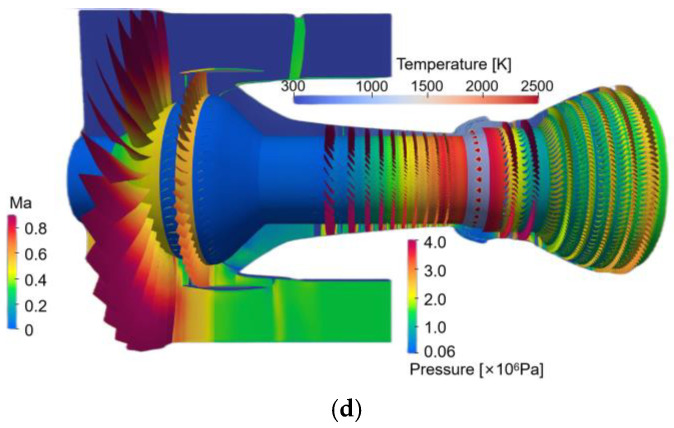
Simulation results of other time steps. (**a**) Velocity, (**b**) Pressure, (**c**) Relative Mach number, (**d**) Mach number, pressure, and temperature.

The flow field results of some other time steps are also shown, as shown in Figure 10. Our solver can conveniently observe the physical quantities of each component.

### 4.3. Implications

Three-dimensional aerodynamic and combustion simulation of the whole turbofan engine is critical to explore the method of engine performance improvement. At present, our team carries out this kind of calculation on the Sunway TaihuLight supercomputer based on the sprayDyMFoam solver. The aimed engine has two spools, a two-stage fan, a 10-stages compressor, a short annular combustor, and seven stages turbine. The total number of grids is 5.1 billion, and 65,336 processors are called to perform this calculation. Performance data demonstrate that the parallel efficiency is 45.59%, the peak computing capacity is 4.25 million cores, and sustained computation is up to 1384.92 DP-GFLOP/s. The simulation flow and combustion results agree well with the experimental data.

We provide a large-scale, detailed, multidisciplinary, and full-engine simulation by integrating advanced physics-based propulsion modeling with high-performance computing and communications technologies.

(a) High-Confidence Computing

The whole circumferential three-dimensional simulation of the entire engine implements the real number of blades, which avoids simulation errors caused by the simplification of the circumferential average of the flow field and the loss of information transmission between components.

The non-simplified full-circumferential simulation can capture the three-dimensional vortex interactions induced by the inlet flow distortion and the shock wave interactions caused by the high speed of the rotor. The simulation results obtained in this way are closer to the physical reality, which is helpful in discovering potential factors affecting performance, providing predictions for avoiding engine surge and rotating stall, and giving suitable guidance for enhancing engine stall margin.

It can also provide the details of the physical field that are difficult to measure in experiments. For instance, the effect of the high-temperature gas behind the combustor on the turbine would be beneficial to further increasing the temperature in the front of the engine turbine and growing the upper limit of the thrust and efficiency.

(b) Capture the Complex Phenomenon

The overall design process of a gas turbine engine is rather complicated. It involves interrelated multidisciplinary and multi-fidelity designs of engine components. The traditional component design process is not always able to understand such complex physical phenomena caused by component interactions. This whole engine simulation could allow designers to capture the complex phenomenon produced by component interactions and have a reasonably accurate prediction of aero-engine performances at the earlier design stage. This simulation would also be capable of predicting strongly coupled multi-component effects, such as compressor-combustor instabilities, combustor-turbine hot-streak migration, and combustion instabilities.

(c) Reduce the Time in Engine Development

Multi-component simulations of the whole engine can obtain the mutual influences and restrictions of component performance and feedback on the matching situation of each component performance synchronously. Synchronous optimization for the performance of each component, rather than independent optimization of the performance of a single component, would be helpful in reducing the design adjustment process, lessening the design iteration, accelerating the design process, and shortening the development cycle.

(d) Reduce the cost

Numerical simulation of the whole engine can effectively replace some of the whole engine experiments and reduce the tests and hardware builds. Further, it can efficiently lessen the cost, fuel consumption, greenhouse gases, and harmful gas emissions.

## 5. Conclusions

1. The efficient implicit solver ‘sprayDyMFoam’ for an unstructured mesh developed in this paper can be effectively applied to the simulation of full three-dimensional flow and combustion on the whole aeroengine, and the performance of the whole machine can match well with the test.
2. An efficient mesh generation method is adopted by transplanting an in-house parallel unstructured meshing code onto Sunway TaihuLight, and the practices have proved the ability to generate billions of mesh elements in dozens of minutes.
3. By adjusting the droplet atomization model, the rules of droplet motion are optimized, and the conflict between large-scale parallelization and the Lagrangian module can be solved. Meanwhile, the adjustment of the PIMPLE algorithm in aerodynamic solution also improves the solution accuracy.
4. The traditional parallel communication mechanism of AMI boundary processing is optimized, which effectively solves the parallel bottleneck of AMI and improves the calculation efficiency.
5. The research carried out in this paper can be applied in high-confidence computing, the complex phenomenon capturing, and time and cost reduction in aeroengine design.

## Figures and Tables

**Table 1:** The comparison of the state of the art [15,16,17,18].

Work	Cores	Mesh Type	Mesh Size	Scenario
1	32,768	Unstructured	2.6 B	Industry
2	65,535	Cartesian	500 M	Scientific
3	65,536	Cartesian	501 M	Scientific
4	1024	Unstructured	24 M	Industry
5	1400	Unstructured	19 M	Industry
6	512	Unstructured	120 M	Industry
Our	65,536	Unstructured	5 B	Scientific

**Table 2:** Efforts on PDE Solvers Nominated for Gordon Bell Prize.

Application	Method	Architecture	Mesh
CFD [21]	Implicit	Multi-core	Unstructured
Atmosphere [22]	Implicit	Multi-core	Structured
Bone Mechanics [23]	Implicit	Multi-core	Unstructured
Phase Field [24]	Explicit	Many-core	Structured
Cloud Cavitation [25]	Explicit	Multi-core	Structured
Earth Mantle [26]	Implicit	Multi-core	Unstructured
Atmosphere [27]	Implicit	Many-core	Structured

**Table 3:** The mesh element counts.

Cases	Mesh Element Number N_E_
CASE-1	80 million
CASE-2	640 million
CASE-3	5.1 billion

**Table 4:** The weak scaling results predicted by the sprayDyMFoam solver on the Sunway TaihuLight.

Mesh	Ncores	*T_N_*	Weak Scaling	DP-GFLOP/s
CASE-1	8320	1.0000	100.00%	6.30
CASE-2	66,560	1.0021	99.79%	50.28
CASE-3	532,480	1.0613	94.22%	379.76

**Table 5:** The strong scaling results predicted by the sprayDyMFoam solver on the Sunway TaihuLight.

Mesh	Ncores	Strong Scaling	DP-GFLOP/s
CASE-1	8320	100.00%	6.30
CASE-1	16,640	99.48%	12.53
CASE-1	33,280	95.10%	23.96
CASE-1	66,560	90.08%	45.39
CASE-2	66,560	100.00%	50.28
CASE-2	133,120	96.95%	97.49
CASE-2	266,240	87.12%	175.20
CASE-2	532,480	77.30%	310.92
CASE-3	532,480	100.00%	379.76
CASE-3	1,064,960	91.43%	694.42
CASE-3	2,129,920	74.23%	1127.57
CASE-3	4,259,840	45.59%	1384.92

## Data Availability

The numerical data used to support the findings of this study are included within the article.

## References

[1] Skira C.A. (2003). Reducing Military Aircraft Engine Development Cost through Modeling and Simulation.

[2] Wang H., Liu B., Mao X., Zhang B., Yang Z. (2022). Combined flow control strategy investigation for corner separation and mid-span boundary layer separation in a high-turning compressor cascade. Entropy.

[3] Singh T.S., Rajak U., Dasore A., Muthukumar M., Verma T.N. (2021). Performance and ecological parameters of a diesel engine fueled with diesel and plastic pyrolyzed oil (PPO) at variable working parameters. Environ. Technol. Innov..

[4] Potier L., Duchaine F., Cuenot B., Saucereau D., Pichillou J. (2022). Prediction of Wall Heat Fluxes in a Rocket Engine with Conjugate Heat Transfer Based on Large-Eddy Simulation. Entropy.

[5] Kesharvani S., Dwivedi G., Verma T.N., Verma P. (2023). The experimental investigation of a diesel engine using ternary blends of algae biodiesel, ethanol and diesel fuels. Energies.

[6] Medic G., Kalitzin G., You D., van der Weide E., Alonso J., Pitsch H. Integrated rans/les computations of an entire gas turbine jet engine. Proceedings of the 45th AIAA Aerospace Sciences Meeting and Exhibit.

[7] Turner M. Lessons learned from the ge90 3-d full engine simulations. Proceedings of the 48th AIAA Aerospace Sciences Meeting Including the New Horizons Forum and Aerospace Exposition.

[8] Wang F. (2013). Whole Aero-Engine Meshing and CFD Simulation. Ph.D. Dissertation.

[9] Krivcov A.V., Shabliy L.S., Baturin O.V. (2014). Gas-dynamic modeling of gas turbine engine components collaborative workflow. Open Mech. Eng. J..

[10] Teixeira M., Romagnosi L., Mezine M., Baux Y., Anker J., Claramunt K., Hirsch C. (2018). A methodology for fully-coupled cfd engine simulations, applied to a micro gas turbine engine. Proceedings of the Turbo Expo: Power for Land, Sea, and Air.

[11] Pérez Arroyo C., Dombard J., Duchaine F., Gicquel L., Odier N., Exilard G., Richard S., Buffaz N., Démolis J. (2020). Large-eddy simulation of an integrated high-pressure compressor and combustion chamber of a typical turbine engine architecture. Proceedings of the ASME Turbo Expo 2020: Turbomachinery Technical Conference and Exposition.

[12] Zhang J., Wei G., Huang W. (2020). Three-dimensional simulation of a core engine. Gas Turbine Exp. Res..

[13] Weller H.G., Tabor G., Jasak H., Fureby C. (1998). A tensorial approach to computational continuum mechanics using object-oriented techniques. Comput. Phys..

[14] Tiwari C., Verma T.N., Dwivedi G., Verma P. (2023). Energy-exergy analysis of diesel engine fueled with microalgae biodiesel-diesel blend. Appl. Sci..

[15] Boileau M., Staffelbach G., Cuenot B., Poinsot T., Bérat C. (2008). Les of an ignition sequence in a gas turbine engine. Combust Flame.

[16] Moriai H., Hori K., Kurose R., Komori S. Large-eddy simulation of spray combution in a sector combustor for regional jet aircraft engine-effect of double-wall liner on nox formation. Proceedings of the Ninth International Symposium on Turbulence and Shear Flow Phenomena.

[17] Moureau V., Domingo P., Vervisch L. (2011). Design of a massively parallel CFD code for complex geometries. C. R. Mécanique.

[18] Bisetti F., Attili A., Pitsch H. (2014). Advancing predictive models for particulate formation in turbulent flames via massively parallel direct numerical simulations. Philos. Trans. R. Soc. A.

[19] Tachibana S., Saito K., Yamamoto T., Makida M., Kitano T., Kurose R. (2015). Experimental and numerical investigation of thermo-acoustic instability in a liquid-fuel aero-engine combustor at elevated pressure: Validity of large-eddy simulation of spray combustion. Combust Flame.

[20] Shen Y., Zhang K., Duwig C. (2022). Investigation of wet ammonia combustion characteristics using LES with finite-rate chemistry. Fuel.

[21] Mirin A.A., Cohen R.H., Curtis B.C., Dannevik W.P., Dimits A.M., Duchaineau M.A., Eliason D.E., Schikore D.R., Anderson S.E., Porter D.H. Very high resolution simulation of compressible turbulence on the IBM-SP system. Proceedings of the 1999 ACM/IEEE Conference on Supercomputing.

[22] Shingu S., Takahara H., Fuchigami H., Yamada M., Tsuda Y., Ohfuchi W., Sasaki Y., Kobayashi K., Hagiwara T., Habata S.I. A 26.58 Tflops global atmospheric simulation with the spectral transform method on the Earth Simulator. Proceedings of the 2002 ACM/IEEE Conference on Supercomputing.

[23] Adams M.F., Bayraktar H.H., Keaveny T.M., Papadopoulos P. Ultrascalable implicit finite element analyses in solid mechanics with over a half a billion degrees of freedom. Proceedings of the 2004 ACM/IEEE Conference on Supercomputing.

[24] Shimokawabe T., Aoki T., Takaki T., Endo T., Yamanaka A., Maruyama N., Nukada A., Matsuoka S. Peta-scale phase-field simulation for dendritic solidification on the tsubame 2.0 supercomputer. Proceedings of the 2011 International Conference for High Performance Computing, Networking, Storage and Analysis.

[25] Rossinelli D., Hejazialhosseini B., Hadjidoukas P., Bekas C., Curioni A., Bertsch A., Futral S., Schmidt S.J., Adams N.A., Koumoutsakos P. 11 p-op/s simulations of cloud cavitation collapse. Proceedings of the International Conference on High Performance Computing, Networking, Storage and Analysis.

[26] Rudi J., Malossi A.C.I., Isaac T., Stadler G., Gurnis M., Staar P.W., Ineichen Y., Bekas C., Curioni A., Ghattas O. An extreme-scale implicit solver for complex pdes: Highly heterogeneous flow in earth’s mantle. Proceedings of the International Conference for High Performance Computing, Networking, Storage and Analysis.

[27] Yang C., Xue W., Fu H., You H., Wang X., Ao Y., Liu F., Gan L., Xu P., Wang L. 10m-core scalable fully-implicit solver for nonhydrostatic atmospheric dynamics. Proceedings of the International Conference for High Performance Computing, Networking, Storage and Analysis.

[28] Fang J., Fu H., Zhao W., Chen B., Zheng W., Yang G. swdnn: A library for accelerating deep learning applications on sunway taihulight. Proceedings of the 2017 IEEE International Parallel and Distributed Processing Symposium (IPDPS).

[29] Liu H., Ren H., Gu H., Gao F., Yang G. (2020). Unat: Unstructured acceleration toolkit on sw26010 many-core processor. Eng. Comput..

[30] Adams M., Brezina M., Hu J., Tuminaro R. (2003). Parallel multigrid smoothing: Polynomial versus gauss–seidel. J. Comput. Phys..

[31] Scales J.A. (1989). On the use of conjugate gradient to calculate the eigenvalues and singular values of large, sparse matrices. Geophys. J. Int..

[32] Ghysels P., Vanroose W. (2014). Hiding global synchronization latency in the preconditioned conjugate gradient algorithm. Parallel Comput..

[33] Chen J., Zhao D., Zheng Y., Xu Y., Li C., Zheng J. (2017). Domain decomposition approach for parallel improvement of tetrahedral meshes. J. Parallel Distr. Com..

[34] Gardner W. (1979). Energy Efficient Engine Flight Propulsion System Preliminary Analysis and Design Report.

[35] Sharma O., Kopper F., Knudsen L., Yustinich J. (1982). Energy Efficient Engine: Low-Pressure Turbine Subsonic Cascade Component Development and Integration Program.

[36] Both A., Mira D., Lehmkuhl O. (2022). Evaporation of volatile droplets subjected to flame-like conditions. Int. J. Heat Mass Tran..

[37] Zhang K., Dybe S., Shen Y., Schimek S., Paschereit C.O., Duwig C. (2020). Experimental and numerical investigation of ultra-wet methane combustion technique for power generation. Proceedings of the Turbo Expo: Power for Land, Sea, and Air.

[38] Amdahl G.M. Validity of the single processor approach to achieving large scale computing capabilities. Proceedings of the Spring Joint Computer Conference.

[39] Gustafson J.L. (1988). Reevaluating amdahl’s law. Commun. ACM.

